# Common variants in the *SLC28A2* gene are associated with serum uric acid level and hyperuricemia and gout in Han Chinese

**DOI:** 10.1186/s41065-018-0078-0

**Published:** 2019-01-16

**Authors:** Zhaowei Zhou, Zhiqiang Li, Can Wang, Xinde Li, Xiaoyu Cheng, Changgui Li, Yongyong Shi

**Affiliations:** 1 Bio-X Institutes, Key Laboratory for the Genetics of Developmental and Neuropsychiaric Disorders (Ministry of Education), Shanghai Jiao Tong University, No. 1954 Huashan Road, Shanghai, 200030 People’s Republic of China; 2 Biomedical Sciences Institute, the Qingdao Branch of SJTU Bio-X Institutes, Qingdao University, Qingdao, 266003 People’s Republic of China; 3 Qingdao Key Laboratory of Gout, The Affiliated Hospital of Qingdao University, Qingdao, 266003 People’s Republic of China; 4 Shandong Provincial Key Laboratory of Metabolic Disease, The Affiliated Hospital of Qingdao University, Qingdao, 266003 People’s Republic of China; 5 Metabolic Disease Institute, Qingdao University, Qingdao, 266003 People’s Republic of China; 6 The Department of Endocrinology and Metabolism, The Affiliated Hospital of Qingdao University, No.16 Jiangsu Road, Qingdao, 266003 People’s Republic of China

**Keywords:** Serum uric acid (SUA), Hyperuricemia (HUA), Gout, *SLC28A2*, Polymorphisms

## Abstract

**Background:**

Serum uric acid (SUA), hyperuricemia (HUA) and gout are complex traits with relatively high heritability. This study aims to identify whether a candidate gene, *SLC28A2*, exerts susceptibility for SUA fluctuation and incidence of HUA and gout in the Han Chinese population.

**Results:**

Three sample sets of 1376 gout patients, 1290 long-term HUA subjects (no gout attack) and 1349 normouricemic controls were recruited for this study. Eight polymorphisms in the *SLC28A2* gene were genotyped using the ligase detection reaction-polymerase chain reaction (LDR-PCR) technology. Rs16941238 showed the most significant associations with SUA level (minor allele “A”, BETA = − 13.84 μmol/L, *P* = 0.0041, P_perm_ = 0.0042) and HUA (OR = 0.7734, *P* = 0.0033, P_perm_ = 0.0020), but not with gout (OR = 0.8801, *P* = 0.1315, P_perm_ = 0.1491). Rs2271437 was significantly associated with gout (minor allele “G”, OR = 1.387, *P* = 0.0277, P_perm_ = 0.0288), and was further confirmed in the meta-analysis with the previously published gout GWAS dataset (OR = 1.3221, *P* = 0.0089). Each variant basically conferred consistent OR direction on gout and HUA, compared with the normouricemic control.

**Conclusions:**

Our findings support the associations of the *SLC28A2* gene with the SUA level, the HUA phenotype and gout in Han Chinese.

**Electronic supplementary material:**

The online version of this article (10.1186/s41065-018-0078-0) contains supplementary material, which is available to authorized users.

## Background

Gout is a metabolic disorder and manifests by a broad spectrum of clinical features including severe and episodic arthritis attack, chronic polyarthritis, palpable tophi and related kidney injuries, which are induced by elevated serum uric acid (SUA) concentrations and consequent deposition of supersaturated urate crystals. Uric acid (UA), the final metabolite of either dietary or endogenous purines, is much higher in humans than in other mammals due to the urate oxidase inactivity resulting from mutational silence during hominoid evolution [1] as well as the effective reabsorption mechanisms mediated by urate transporters expressed in kidney [2]. Thus, humans are more susceptible to exceed the reference range of SUA concentrations and suffer from HUA, defined as SUA > 420 μmol/L in men and postmenopausal women or SUA > 360 μmol/L in premenopausal women [3]. High SUA is the most causative factor of gout and the higher the SUA, the higher the gout incidence [4]. Besides, both HUA and gout always cluster with a variety of comorbidities including obesity, insulin resistance, type 2 diabetes, hypertension and other cardiovascular diseases [4, 5]. Due to the ever aging population worldwide, the changing of dietary structure and increased incidence of comorbidities etc., the prevalence of HUA and gout have been climbing dramatically and become major public healthcare issues [4].

SUA and HUA are complex traits and disorders with individual heritability reaching up to 70% [6] and 60% [7], respectively. Gout shows evident aggregation within families and reported a heritability of 35.1% in men and 17.0% in women, indicating the importance of genetics as well [8]. Thus, detecting genetic determinants associated with SUA, HUA and gout is a key step in exploring the pathogenesis and potential therapeutic targets of the related disorders. Extensive genetic studies, especially large-scale genome-wide association studies (GWAS), have identified dozens of susceptibility genes [4]. Despite, all these genetic determinants can only explain < 10% variations for the disorders, indicating additional loci to be found [9].

One-third of UA produced per day are acquired from dietary purines [10]. Thus, daily consumed food may affect the size of purines pool in the circulation and consequently cause UA overproduction. There have been multiple epidemiological investigations reporting that excessive consumption of purine-rich food was positively correlated with SUA level and gout incidence [11, 12]. Parallel, constraint of purine-rich food consumption was found to lower SUA level and alleviate gout flares [13]. Nucleosides absorption across luminal membrane of intestine into enterocytes is controlled by concentrative nucleoside transporters (CNTs), encoded by solute carrier (SLC) 28 family genes [14]. CNT2, encoded by the *SLC28A2* gene, is expressed in luminal membrane of intestine and mediates efficient transport of dietary purine nucleosides and nucleoside analog ribavirin [14, 15]. Previously, a GWAS of SUA in a relatively isolated population of European descent from the Adriatic coast of Croatia identified the *SLC28A2* gene to be suggestive in relation to SUA concentrations (*P* < 5 × 10^− 6^) [16]. In our previous GWAS with Han Chinese samples of clinically diagnosed gout patients and healthy individuals, we did not find significant associations between the *SLC28A2* gene and gout at the discovery stage (P_GWAS stage_ > 0.05) [17].

The current study is designed to investigate relationships between *SLC28A2* polymorphisms and SUA concentrations, HUA, as well as clinically ascertained gout in the Han Chinese population with additional sample sets. Then, by performing a meta-analysis combining results obtained in the present study and that acquired from our previous GWAS, we attempt to further examine the relationship between the *SLC28A2* gene and gout.

## Results

Clinical characteristics of all participants were summarized in Table 1. The mean age of gout onset was 44 years old and the proportion of tophus was 21.7%. The mean SUA value among among gout, HUA and normouricemic control was 465.13 μmol/L, 475.55 μmol/L and 284.17 μmol/L, respectively. Since lots of samples were not recorded with comorbidities such as kidney stones, hypertension, diabetes, coronary heart disease and hyperlipidemia, we presented the specific numbers of the recorded and did not perform statistical analysis.

**Table 1:** Clinical characteristics of gout, HUA and normouricemic control dataset

Index	Gout	HUA	Control
Age (year)	49.72 ± 13.28	49.75 ± 15.45	57.58 ± 14.36
Age of gout onset (year)	44.00 ± 12.91	–	–
SUA (μmol/L)	465.13 ± 115.00	475.55 ± 56.06	284.17 ± 52.05
Tophus (%)	21.70% (199/1116)	–	–
Kidney stones (%)	17.7% (178/1007)	–	–
Hypertension (%)	38.1% (458/1201)	72.4% (218/301)	24.8% (75/303)
Diabetes (%)	7.9% (96/1215)	25% (32/128)	14.5% (44/303)
Coronary heart disease (%)	7.6% (91/1201)	38.8% (59/152)	12.6% (37/294)
Hyperlipidemia (%)	28.5% (126/442)	78.7% (74/94)	–

Age, age of gout onset and SUA value are continuous variables and are expressed by mean ± standard deviation (SD). Comorbidities including tophus, kidney stones, hypertension, diabetes, coronary heart disease and hyperlipidemia are categorical variables and are expressed by percentages. Since lots of samples were not recorded with these comorbidities, we presented the specific numbers of the recorded in brackets and did not perform statistical analysis. - denotes not acquired

Genotypes of the forty duplicates were 100% identical, and all the SNPs passed the HWE tests (*P* > 0.0063, seen in Table 2). Allelic frequencies and association results are displayed in Table 2. In the gout versus control cohort: only rs2271437 showed significant association (minor allele “G”, OR = 1.387, *P* = 0.0277, P_perm_ = 0.0288). In the HUA versus control cohort: rs16941238 showed the most significant association (minor allele “A”, OR = 0.7734, *P* = 0.0033, P_perm_ = 0.0020); the other two SNPs, rs2413769 and rs11639349, showed significant associations too (minor allele “T”, OR = 0.8406, *P* = 0.0184, P_perm_ = 0.0172 and minor allele “T”, OR = 1.363, *P* = 0.0171, P_perm_ = 0.0219, respectively). In the gout versus HUA cohort: no significant difference was detected except for rs11639349 (minor allele “T”, OR = 0.7419, *P* = 0.0213, P_perm_ = 0.0211). In the combined HUA + GOUT versus control analysis, all SNPs showed the same effect direction in the separate HUA and gout analysis and two SNPs, rs2413769 and rs16941238 reached the statistical significance (OR = 0.8645, *P* = 0.0202, P_perm_ = 0.0324 and OR = 0.8279, *P* = 0.0101, P_perm_ = 0.0140, respectively). Considering that gout patients might be prescribed with urate lowering therapy that might affect SUA concentrations, we evaluated the effect of each SNP on SUA only in the HUA cohort combined with the normouricemic controls (seen in Table 3). Rs16941238 showed the most significant association with SUA level (minor allele “A”, BETA = − 13.84 μmol/L, *P* = 0.0041, P_perm_ = 0.0042) and rs2413769 was the second in significance (minor allele “T”, BETA = − 10.28 μmol/L, *P* = 0.0120, P_perm_ = 0.0143). Then, we present the frequency and mean SUA value for each genotype of the two most significant loci (rs16941238 and rs2271437) among gout, HUA and normouricemic controls, respectively (Additional file 1: Table S2).

**Table 2:** Results of the seven SNPs in gout versus control cohort, HUA versus control cohort, gout versus HUA cohort and gout+HUA versus control cohort

SNP	A1	F_Gout	F_HUA	F_Gout+HUA	F_Ctrl	HWE P	Gout vs Ctrl OR	Gout vs Ctrl P	Gout vs Ctrl Pperm	HUA vs Ctrl OR	HUA vs Ctrl P	HUA vs Ctrl Pperm	Gout vs HUA OR	Gout vs HUA P	Gout vs HUA Pperm	Gout+HUA vs Ctrl OR	Gout+HUA vs Ctrl P	Gout+HUA vs Ctrl Pperm
rs11854484	T	0.0480	0.0543	0.0510	0.0541	0.576	0.8822	0.3061	0.3333	1.003	0.9810	1	0.879	0.2985	0.3256	0.9402	0.5567	0.8571
rs2413775	T	0.1890	0.1843	0.1867	0.1998	0.754	0.9321	0.3088	0.3256	0.9033	0.1503	0.2419	1.032	0.6584	0.75	0.9179	0.1552	0.2344
rs2413769	T	0.1701	0.1624	0.1664	0.1868	0.465	0.8883	0.1005	0.0914	0.8406	**0.0184**	**0.0172**	1.059	0.4440	0.5625	0.8645	**0.0202**	**0.0324**
rs1060896	A	0.0493	0.0574	0.0532	0.0542	0.980	0.9056	0.4176	0.4	1.063	0.6112	0.6667	0.852	0.1899	0.2273	0.9813	0.8572	1
rs2271437	G	0.0418	0.0368	0.0394	0.0308	0.801	1.387	**0.0277**	**0.0288**	1.205	0.2236	0.35	1.145	0.3439	0.5	1.298	0.0499	0.0667
rs11639349	T	0.0422	0.0557	0.0487	0.0415	0.067	1.016	0.9077	0.8571	1.363	**0.0171**	**0.0219**	0.7419	**0.0213**	**0.0211**	1.181	0.1510	0.1589
rs16941238	A	0.1170	0.1043	0.1108	0.1301	0.060	0.8801	0.1315	0.1491	0.7734	**0.0033**	**0.0020**	1.142	0.1337	0.1328	0.8279	**0.0101**	**0.0140**
rs765787	C	0.3416	0.3433	0.3424	0.3340	0.008	1.037	0.54	0.7	1.045	0.462	0.75	0.9921	0.8927	1	1.041	0.4385	0.5625

A1, minor allele for the whole sample, namely the effect allele (the one that the reported OR correlates with); F_Gout, frequency of A1 in the gout cohort; F_HUA, frequency of A1 in the HUA cohort; F_Gout+HUA, frequency of A1 in the Gout+HUA cohort; F_Ctrl, frequency of A1 in the control cohort; OR, odds ratio; P, *P* value; Pperm, empirical permutation p values were permutated randomly with up to 1,000,000 trials. Pperm< 0.05 as the significance threshold and significant *p* values in bold

**Table 3:** Results of the seven SNPs with SUA concentrations in combined HUA and control dataset

CHR	SNP	A1	BETA	P	Pperm
15	rs11854484	T	−3.39	0.6125	1
15	rs2413775	T	−3.419	0.3859	0.6154
15	rs2413769	T	−10.28	**0.0120**	**0.0143**
15	rs1060896	A	−1.013	0.8788	1
15	rs2271437	G	13.89	0.1014	0.0928
15	rs11639349	T	9.105	0.2012	0.2381
15	rs16941238	A	−13.84	**0.0041**	**0.0042**
15	rs765787	C	0.0544	0.9869	1

Shown are the effect size (BETA) and P value for the association of each SNP with SUA concentrations. The unit for BETA isμmol/L. Effect estimates result from additive linear regression on Z-scores of SUA concentrations. Pperm< 0.05 as the significance threshold and significant *p* values in bold

**Fig. 1:**
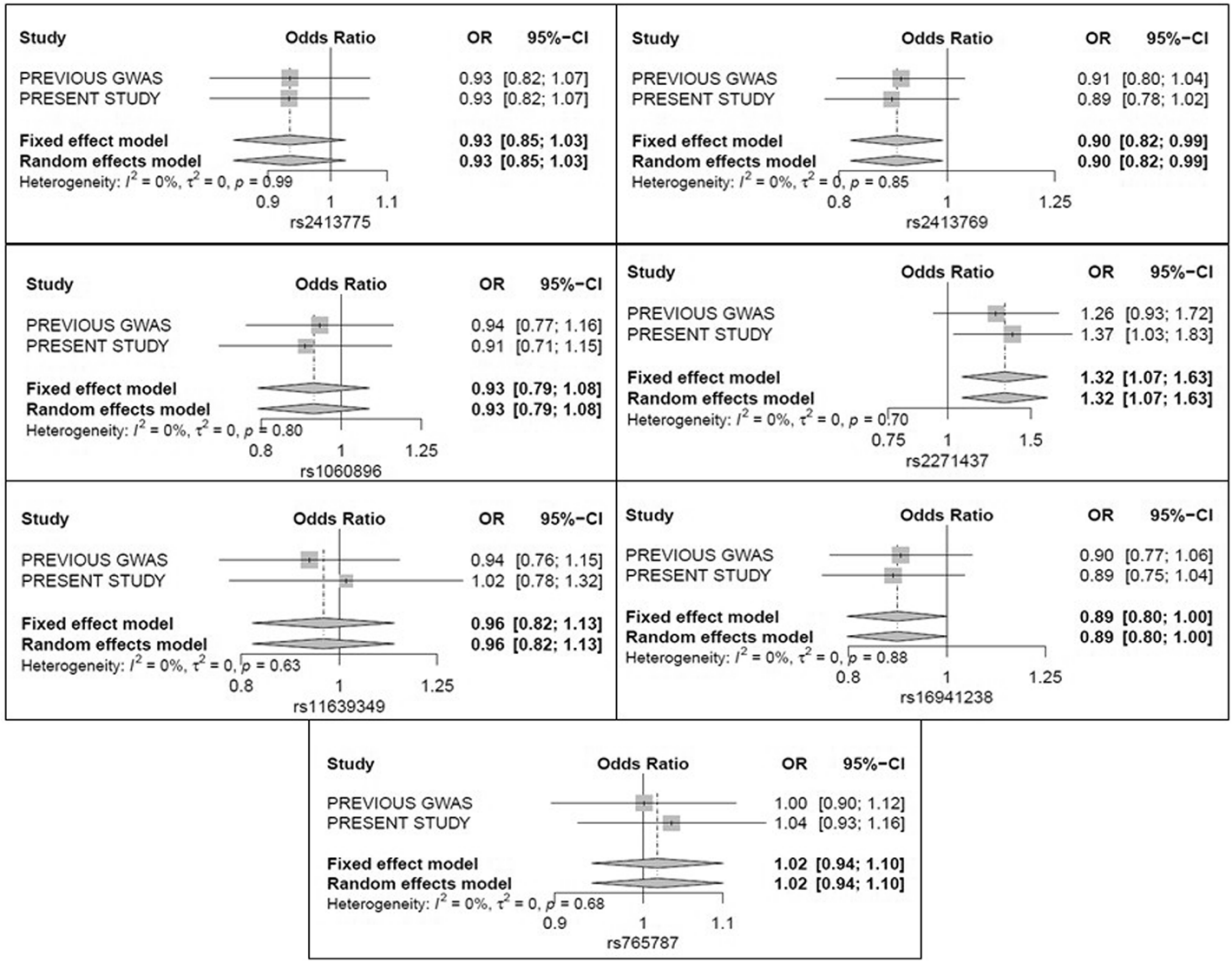
Forest plot of the association between the seven SNPs and gout

For the meta-analysis, there are seven SNPs having both data in the current study and the previous reported GWAS [17]. None of the seven SNPs (rs2413775, rs1060896, rs2271437, rs2413769, rs16941238, rs11639349 and rs765787) reached the nominal significance in the discovery stage of gout (*P* > 0.05), but the OR directions were basically in accordance with the present study (seen in Fig. 1 and Table 2). By meta-analysis, we noted two SNPs showed significant results (rs2413769, OR = 0.9009, *P* = 0.0328; rs2271437, OR = 1.3221, *P* = 0.0089).

**Fig. 2:**
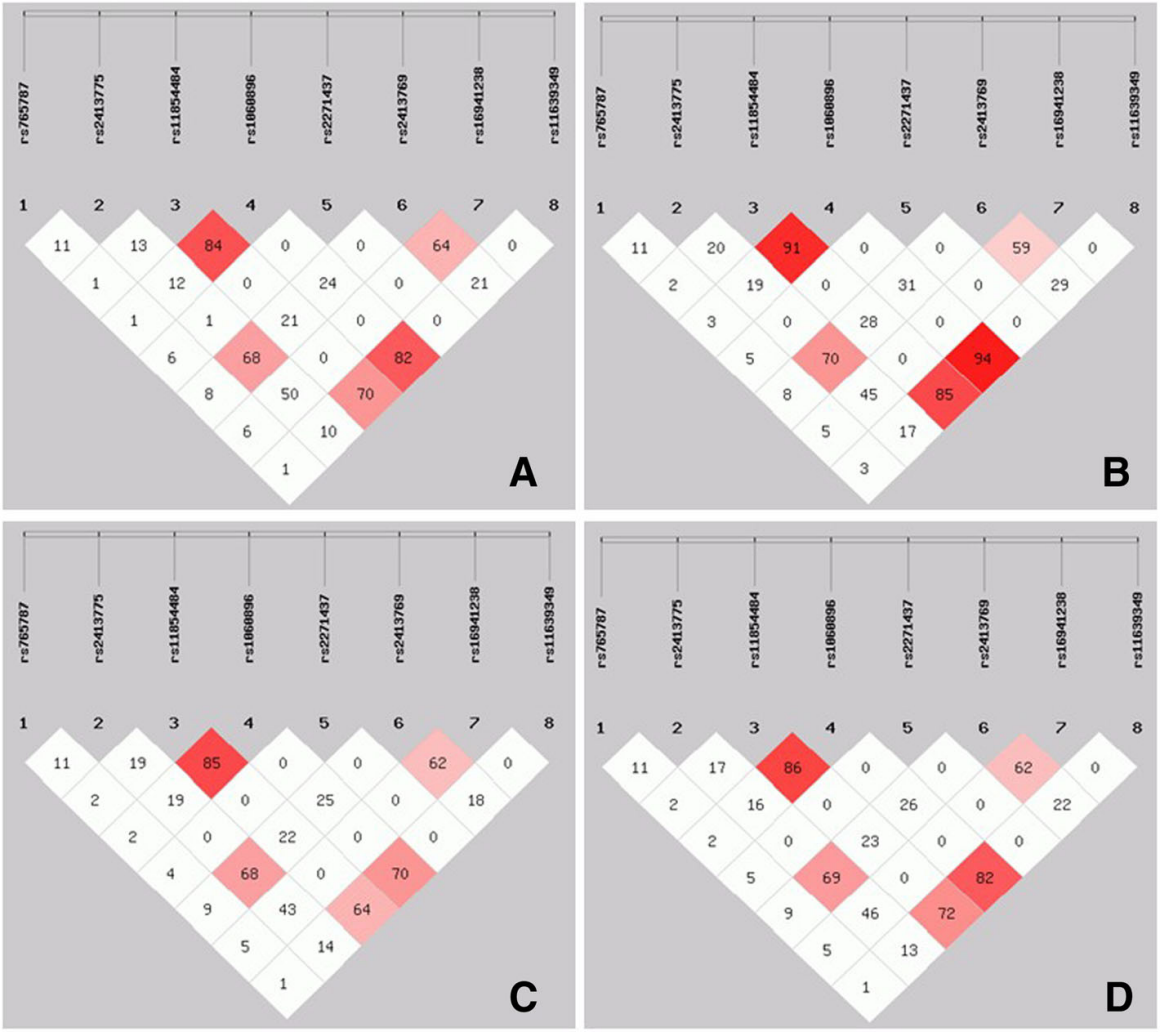
Linkage disequilibrium plot of the eight SNPs in the samples of gout patients (**a**), HUA cohort (**b**), normouricemic controls (**c**) and the overall (**d**)

At last, we estimated the linkage disequilibrium (r^2^ > 0.95) among the eight SNPs in gout patients, HUA cohort, normouricemic controls and the overall, respectively. Haplotype distributions were similar in the different sample sets and no specific haplotype block was identified, as shown in Fig. 2.

## Discussion

The *SLC28A2* gene encodes the transport protein of CNT2, which is mainly expressed in luminal membrane of intestine [18] and transports preferentially purine nucleosides including adenosine [18]. Excessive circulating adenosine can be degraded into UA in human body [19]. On the other hand, adenosine is a kind of mediator to suppress inflammatory and immune responses through binding to surface receptor of A2AR. Mechanism studies have confirmed that A2AR activation attenuates progression of experimental arthritis by inhibiting TNF-α [20] and IL-1β production [21], both of which are the key pro-inflammatory cytokines in the pathogenesis of inflammation flare. There may exit such a phenomenon that risk allele carriers of the *SLC28A2* gene are more prone to HUA but not gout attack. Concordantly, we found rs11639349 T allele significantly increased susceptibility to HUA (OR = 1.363, *P* = 0.0171, P_perm_ = 0.0219), rather than gout (OR = 1.016, *P* = 0.9077, P_perm_ = 0.8571) while T allele showed protective effect on gout incidence compared to HUA cohort (OR = 0.7419, *P* = 0.0213, P_perm_ = 0.0211). In this study, we identified intron SNPs (rs16941238 and rs2413769) significantly associated with both SUA level and HUA phenotype, and another exonic SNP (rs2271437) associated with gout in the *SLC28A2* gene. Nevertheless, the elaborate biological mechanisms caused by the *SLC28A2* variants remain unclear.

In a SUA GWAS using European individuals, rs765787 locus which is in high linkage disequilibrium with the *SLC28A2* gene was identified to be marginally associated with SUA fluctuation [16]. In our study, this site failed to reach statistical significance even in the meta-analysis. Rs2413775 is located in 5’-UTR region and T allele was found to enhance the *SLC28A2* gene expression in comparison with A allele [22]. However, we found no significant association for this variant in the present study. Its MAF (=0.198) was as the same of HapMap-HCB (=0.205) and presents significant lower value than HapMap-CEU (=0.775), suggesting the existence of genetic heterogeneity among populations. Overall, the allele frequencies of these selected SNPs are almost consistent with those of HapMap-HCB but present significant lower values than HapMap-CEU, except for rs2271437 and rs765787 (seen in Table 2 and Additional file 1: Table S1).

The intron variant rs16941238 showed the most significant associations with both SUA concentrations and HUA phenotype, with the minor allele conferring the same effect tendency on decreasing SUA (BETA< 0, *P* < 0.05) and less predisposing to HUA status (OR < 1, P < 0.05) but not with gout even in the meta-analysis (OR < 1, *P* > 0.05). Other genetic studies also manifest this phenomenon that significant loci associated with SUA concentrations, most of which were intron variants, may not reach significance with gout but indeed increase or decrease the risk along with the same effect direction [9]. Although the significant polymorphisms locate in the intron regions, there may be effects on the gene splicing, transcription factor binding and mRNA degradation. Less common variant rs2271437 produces an amino acid change from Leucine to Tryptophan at position 163 (p.L163 W) and was observed to exert much higher risk to gout development (OR > 1, *P* < 0.05) which needs further characterization for biological function in the future. Lots of researches have focused on functionally characterizing *SLC28A2* functional variants in mode cells and discovered that amino acid residues in transmembrane (TM) 7–9 region were most important for substrate selectivity and transport activity: Glycine at position 313 (TM7) and Threonine at position 347 (TM8) were within the substrate translocation channel and determined the substrate selectivity for purines [23]; substitution of Serine for original Phenylalanine at 355 led to a sharp decrease in uridine selectivity with no change for inosine and ribavirin transport [24]; a more comprehensive study incorporating 96 healthy volunteers of each group from Chinese, Malays and Indians identified 23 variants in total of which the allele frequencies manifested significant ethnic difference and only one variant p.E385K (TM8–9) showed inability to transport inosine and ribavirin [25]. Unfortunately, we did not genotype variants in this region due to their MAF = 0 in HapMap-HCB.

According to the current guidelines for HUA and gout, extreme HUA status (SUA > 9 mg/dL) and clinical gout are both indications of urate lowering therapy (ULT) [26]; lifestyle modification such as restriction of purine-rich food intake is also necessary and adjuvant in management process which will in turn reduce the quality of life and consequent bad compliance [26, 27]. For pharmacologic ULT, the varieties are limited [26, 27], and the target rate of optimal SUA level (< 6 mg/dL) was found to be poor in clinical practice and the adverse side effects were well confirmed [28]. Thus, the development of new medications has been a vibrant field of research. Our findings provide some new clues into the molecular mechanism underlying the disease incidence which needs to be more widely replicated. Notably, many institutions have developed some CNT2 inhibitors exhibiting potent CNT2 inhibitory activity with less side effects and attempted to ameliorate them into clinical applications [29–32].

## Conclusions

In conclusion, we demonstrate the significant associations of intron variants in the *SLC28A2* gene with both SUA level and HUA phenotype, and another exonic variant with gout. Replication studies incorporating multiple ethnic populations and functional research are suggested to further validate the present results.

## Methods

### Study sample set

All subjects were males of Han Chinese origin living in Shandong coastal area. Once selected for the study, all subjects signed informed consent. This research was approved by the Bio-X Ethics Committee of Shanghai Jiao Tong University (approval number: ML18003) and conformed to the principles of the current version of the Declaration of Helsinki [33].

The study sample comprised three sets: 1376 unrelated gout patients, 1290 unrelated HUA subjects and 1349 unrelated normouricemic controls (seen in Table 1). All gout patients were clearly diagnosed by two gout specialists from the Affiliated Hospital of Qingdao University on the basis of gout diagnosis and treatment guidelines developed by American College of Rheumatology (ACR) in 1977 [34]. Those simultaneously suffering from severe complications such as cancer, uremia and myloproliferative disorders were excluded. All HUA subjects and normouricemic controls were collected from field survey by Shandong Provincial Key Laboratory of Metabolic Disease, the Affiliated Hospital of Qingdao University and also interviewed by two gout specialists for further confirmation. The specific inclusion and exclusion criteria were as described previously [17]. All samples did not overlap with those in our previous gout GWAS [17].

### SNPs selection and genotyping

Haploview 4.2 version was used to filter common tag single nucleotide polymorphisms (SNPs) of the *SLC28A2* gene in terms of HapMap Chinese Han in Beijing (HapMap-HCB) and five SNPs including rs11854484, rs2271437, rs9635306, rs2413769 and rs16941238, were chosen with r^2^ > 0.8 filter criteria, and rs9635306 was excluded since it is an entirely conserved site (minor allele frequency (MAF) = 0 in HapMap-HCB). SNP rs1060896 which is located in exon 2 and results in an amino acid change, and rs11639349 which is located in 3’-UTR region, were also included for analysis. The other two SNPs, rs765787 and rs2413775, were selected according to review of literature: rs765787, located in ~ 44 kb upstream of the *SLC28A2* gene, was identified to be marginally associated with SUA concentrations in a GWAS [16]; rs2413775, which is located in 5’-UTR region and T allele enhanced the *SLC28A2* gene expression compared with A allele, which might increase intestinal purine absorption and thus UA overproduction [22]. Other known functional variants, such as p.F355S and p.E385K [24, 25], were not selected due to their MAF = 0 in HapMap-HCB. The SNPs information are displayed in Additional file 1: Table S1. Therefore, eight SNPs in total, rs11854484, rs2271437, rs2413769, rs16941238, rs1060896, rs11639349, rs765787 and rs2413775 were genotyped for this study.

Blood genomic DNA extraction kit (Lifefeng Biotechnology Co., Ltd., Shanghai, China), was applied to draw genomic DNA from peripheral blood samples of all subjects. Genotypes of all the SNPs were determined using the ligase detection reaction-polymerase chain reaction (LDR-PCR) technology [35, 36] in Shanghai Biowing Applied Biotechnology Co., Ltd. (http://www.biowing.com.cn). In order to control the genotyping quality, forty internal positive and negative duplicates were used, and the concordance rate was 100%.

### Statistical analysis

Hardy-Weinberg equilibrium (HWE) was conducted in the control group for each SNP based on goodness-of-fit chi-square test using free-charge SHEsisPlus online platform (http://shesisplus.bio-x.cn/), with α = 0.0063 (0.05/8) [37, 38]. .Pairwise linkage disequilibrium was also analyzed by SHEsisPlus. Logistic regression analysis was applied to test the association of each SNP with qualitative traits (HUA and gout). Effect of each SNP on SUA concentrations was evaluated using linear regression analysis. The effect size of each SNP was embodied by odds ratio (OR) in logistic regression analysis or BETA value in linear regression analysis. Regression analyses were made by using PLINK [39]. Permutation testing was applied to correct the multiple tests effects and estimate empirical *P* values. Empirical *P* < 0.05 was set as statistical significance.

### Meta-analysis

In our previous gout GWAS, two genotyped SNPs (rs1060896, rs2413769) and additional five imputed SNPs (rs765787, rs2413775, rs2271437, rs16941238, rs11639349) [17] mapping to the *SLC28A2* gene were analyzed. Then, we combine this dataset with the results obtained from the present study (1290 gout patients versus 1349 normouricemic controls) for a meta-analysis. Fixed-effect model was used to calculate combined ORs if there was no significant heterogeneity, otherwise, the random-effect model was used. Heterogeneity was assessed by Q statistic and quantified by I^2^ statistics. Meta-analysis was performed using PLINK. The significance threshold was defined as *P* < 0.05.

## Additional files

Additional file 1: **Table S1.** The SNPs information in the analysis. **Table S2.** The frequency and mean SUA value for each genotype of rs16941238 and rs2271437 among gout, HUA and normouricemic controls, respectively. (DOCX 22 kb)

## Abbreviations

ACR: American College of Rheumatology
CEU: Utah residents with Northern and Western Europe
CHR: Chromosome
CNT: Concentrative nucleoside transporter
Ctrl: Control
F: Frequency
GWAS: Genome-wide association study
HCB: Han Chinese in Beijing
HUA: Hyperuricemia
HWE: Hardy-Weinberg equilibrium
OR: Odds ratio
P: *P* value
Pperm: Empirical permutation *p* values
SD: Standard deviation
SLC: Solute carrier
SNP: Single nucleotide polymorphism
SUA: Serum uric acid
TM: Transmembrane
ULT: Urate lowering therapy
