# Supplementary material for: Common variants in the SLC28A2 gene are associated with serum uric acid level and hyperuricemia and gout in Han Chinese
Source: Hereditas. 2019 Jan 16;156:4. doi: 10.1186/s41065-018-0078-0 (PMC6335706; doi:10.1186/s41065-018-0078-0)
Supplement: Supplementary file 1 — Table S1. The SNPs information in the analysis. Table S2. The frequency and mean SUA value for each genotype of rs16941238 and rs2271437 among gout, HUA and normouricemic controls, respectively. (DOCX 22 kb) [file 41065_2018_78_MOESM1_ESM.docx]

**Table S1 The SNPs information in the analysis**

| SNP | Position (GRCh38.p2) | | Functional site | Polymorphism | Amino acid change or functional change | MAF*CEU | MAF*HCB |
| --- | --- | --- | --- | --- | --- | --- | --- |
| Rs11854484 | Chr15：45253280 | Exon1 | | C>T | Pro22Leu | - | - |
| Rs2413775 | Chr15：45252090 | 5’-UTR | | A>T | A gain of function | 0.775 | 0.205 |
| Rs2413769 | Chr15：45269670 | Intron14-15 | | G>T | Not sure | 0.819 | 0.122 |
| Rs1060896 | Chr15：45262069 | Exon2 | | C>A | Ser75Arg | 0.665 | 0.049 |
| Rs2271437 | Chr15：45263922 | Exon5 | | T>G | Leu163Trp | 0 | 0.049 |
| Rs11639349 | Chr15：45276062 | 3’-UTR | | C>T | Not sure | 0.664 | 0.049 |
| Rs16941238 | Chr15：45269706 | Intron14-15 | | G>A | Not sure | 0.155 | 0.073 |
| Rs765787 | Chr15：45207849 | ~44kb upstream | | T>C | Not sure | 0.164 | 0.354 |

* collected from NCBI dbSNP database. CEU: Utah residents with Northern and Western European ancestry from the CEPH collection, CHB: Han Chinese in Beijing.

**Table S2 The frequency and mean SUA value for each genotype of rs16941238 and rs2271437 among gout, HUA and normouricemic controls, respectively**

| Groups | Rs2271437 | | | | | | Rs16941238 | | | | | |
| --- | --- | --- | --- | --- | --- | --- | --- | --- | --- | --- | --- | --- |
|  | GG | | GT | | TT | | AA | | AG | | GG | |
|  | Freq | SUA (μmol/L) | Freq | SUA (μmol/L) | Freq | SUA (μmol/L) | Freq | SUA (μmol/L) | Freq | SUA (μmol/L) | Freq | SUA (μmol/L) |
| Gout | 0 | - | 115 | 480.25±108.26 | 1260 | 463.81±115.55 | 11 | 476.80±56.57 | 300 | 459.50±115.97 | 1065 | 466.58±115.20 |
| HUA | 2 | 475.00±62.23 | 91 | 479.72±56.10 | 1197 | 475.24±56.09 | 15 | 466.52±39.03 | 239 | 474.48±58.09 | 1035 | 475.93±55.83 |
| Control | 1 | 308.00±00 | 81 | 290.37±48.18 | 1267 | 283.76±52.30 | 15 | 286.39±45.00 | 321 | 283.28±50.69 | 1013 | 284.42±52.60 |

The SUA level is expressed by mean ± standard deviation (SD). Freq means frequency. - denotes not acquired.
